# Kinetic Analysis of Acanthamoeba castellanii Infected with Giant Viruses Quantitatively Revealed Process of Morphological and Behavioral Changes in Host Cells

**DOI:** 10.1128/spectrum.00368-21

**Published:** 2021-08-25

**Authors:** Sho Fukaya, Masaharu Takemura

**Affiliations:** a Department of Applied Information Engineering, Faculty of Engineering, Suwa University of Science, Chino, Nagano, Japan; b Laboratory of Biology, Institute of Arts and Sciences, Tokyo University of Science, Shinjuku, Tokyo, Japan; c Laboratory of Biology, Graduate School of Mathematics and Science Education, Tokyo University of Science, Shinjuku, Tokyo, Japan; City University of Hong Kong

**Keywords:** acanthamoeba, behavior, giant virus, image analysis, marseillevirus, medusavirus, mimivirus, morphology, pandoravirus

## Abstract

Most virus-infected cells show morphological and behavioral changes, which are called cytopathic effects. Acanthamoeba castellanii, an abundant, free-living protozoan, serves as a laboratory host for some viruses of the phylum *Nucleocytoviricota*—the giant viruses. Many of these viruses cause cell rounding in the later stages of infection in the host cells. Here, we show the changes that lead to cell rounding in the host cells through time-lapse microscopy and image analysis. Time-lapse movies of A. castellanii cells infected with Mimivirus shirakomae, kyotovirus, medusavirus, or Pandoravirus japonicus were generated using a phase-contrast microscope. We updated our phase-contrast-based kinetic analysis algorithm for amoebae (PKA3) and used it to analyze these time-lapse movies. Image analysis revealed that the process leading to cell rounding varies among the giant viruses; for example, *M. shirakomae* infection did not cause changes for some time after the infection, kyotovirus infection caused an early decrease in the number of cells with typical morphologies, and medusavirus and *P. japonicus* infection frequently led to the formation of intercellular bridges and rotational behavior of host cells. These results suggest that in the case of giant viruses, the putative reactions of host cells against infection and the putative strategies of virus spread are diverse.

**IMPORTANCE** Quantitative analysis of the infection process is important for a better understanding of viral infection strategies and virus-host interactions. Here, an image analysis of the phase-contrast time-lapse movies displayed quantitative differences in the process of cytopathic effects due to the four giant viruses in Acanthamoeba castellanii, which were previously unclear. It was revealed that medusavirus and Pandoravirus japonicus infection led to the formation of a significant number of elongated particles related to intercellular bridges, emphasizing the importance of research on the interaction of viruses with host cell nuclear function. Mimivirus shirakomae infection did not cause any changes in the host cells initially, so it is thought that the infected cells can actively move and spread over a wider area, emphasizing the importance of observation in a wider area and analysis of infection efficiency. These results suggest that a kinetic analysis using the phase-contrast-based kinetic analysis algorithm for amoebae (PKA3) reveals the infection strategies of each giant virus.

## INTRODUCTION

*Acanthamoeba*, an abundant and free-living protozoan, is a pathogenic eukaryotic microorganism that causes *Acanthamoeba*-associated keratitis (1). It also serves as a host for some viruses of the phylum *Nucleocytoviricota*, the “giant viruses” (2), including the families *Mimiviridae* and *Marseilleviridae*, pandoraviruses, pithoviruses, molliviruses, and medusavirus (2–8). Healthy *Acanthamoeba* cultured under laboratory conditions adhere to the bottom of the culture flask and move freely on the surface of the flask using pseudopodia. Giant virus infections alter the morphology of infected *Acanthamoeba* cells, and the changes are termed cytopathic effects (CPEs). Because of the free-moving characteristics of *Acanthamoeba*, CPEs can be observed not only in their morphology, but also in their behavior. Various CPEs have been reported, including cell rounding, lysis, aggregation, and cyst formation (8–12). Most of the viruses that infect *Acanthamoeba* cause cell rounding in the late stages of infection, and then some viruses lead to cell lysis (8, 10–13). It has also been reported that tupanvirus (*Mimiviridae*) and hokutovirus (*Marseilleviridae*) cause cell aggregation before cell rounding (9, 10). Kyotovirus, another *Marseilleviridae* family virus, does not cause host cell aggregation (10). Thus, even if the final host cell morphology caused by viral infection is similar, the process leading to that morphology may differ depending on the type of the virus. Hence, elucidation of the morphological and behavioral characteristics in the process of infection is important for studying viral infection strategies and virus-host interactions.

Phase-contrast microscopy is widely used to observe the behavior of living cells because it does not require staining or alteration of cells (14, 15). However, the artifacts called “halo” or “shade-off” that appear in phase-contrast microscopic images hinder automated image analysis (14). In a previous study, we developed an image analysis algorithm named the phase-contrast-based kinetic analysis algorithm for amoebae (PKA3), which quantitatively reveals behavioral characteristics of the host cells in the process of infection with kyotovirus and hokutovirus (16). PKA3 can analyze time-lapse phase-contrast microscopic images of nonlabeled living amoebae and provide output in the form of number, movement, and direction of particles that are considered to be amoebae. To date, only images of Acanthamoeba castellanii cells infected with viruses of the *Marseilleviridae* family have been analyzed using PKA3 (16, 17). However, it can also be used to analyze the behavior of A. castellanii cells infected with giant viruses from other families.

In this study, we updated PKA3 for a more accurate analysis of the cell morphology in high-magnification images and some additional parameters. The new algorithm was used to analyze A. castellanii infected with four types of giant viruses, kyotovirus of the *Marseilleviridae* family (10), Mimivirus shirakomae of the *Mimiviridae* family (18), medusavirus (8), and Pandoravirus japonicus of the *Pandoraviridae* family (19) isolated in Japan. Infection with these viruses is known to cause cell rounding in A. castellanii. Then, A. castellanii infected with *M. shirakomae* undergoes cell lysis, and A. castellanii infected with medusavirus undergoes cyst formation. Therefore, it is unknown whether the host cells infected with these viruses display different morphological and behavioral characteristics during the infection process that lead to cell rounding. In addition, viruses that caused aggregation behavior in host cells, such as hokutovirus (16), were not included in this study. This is because the individual cells could not be identified within the aggregates, and the morphology of a single cell could not be compared with that of the other viruses.

## RESULTS

### Analyzed images of A. castellanii infected with the four giant viruses.

**FIG 1:**
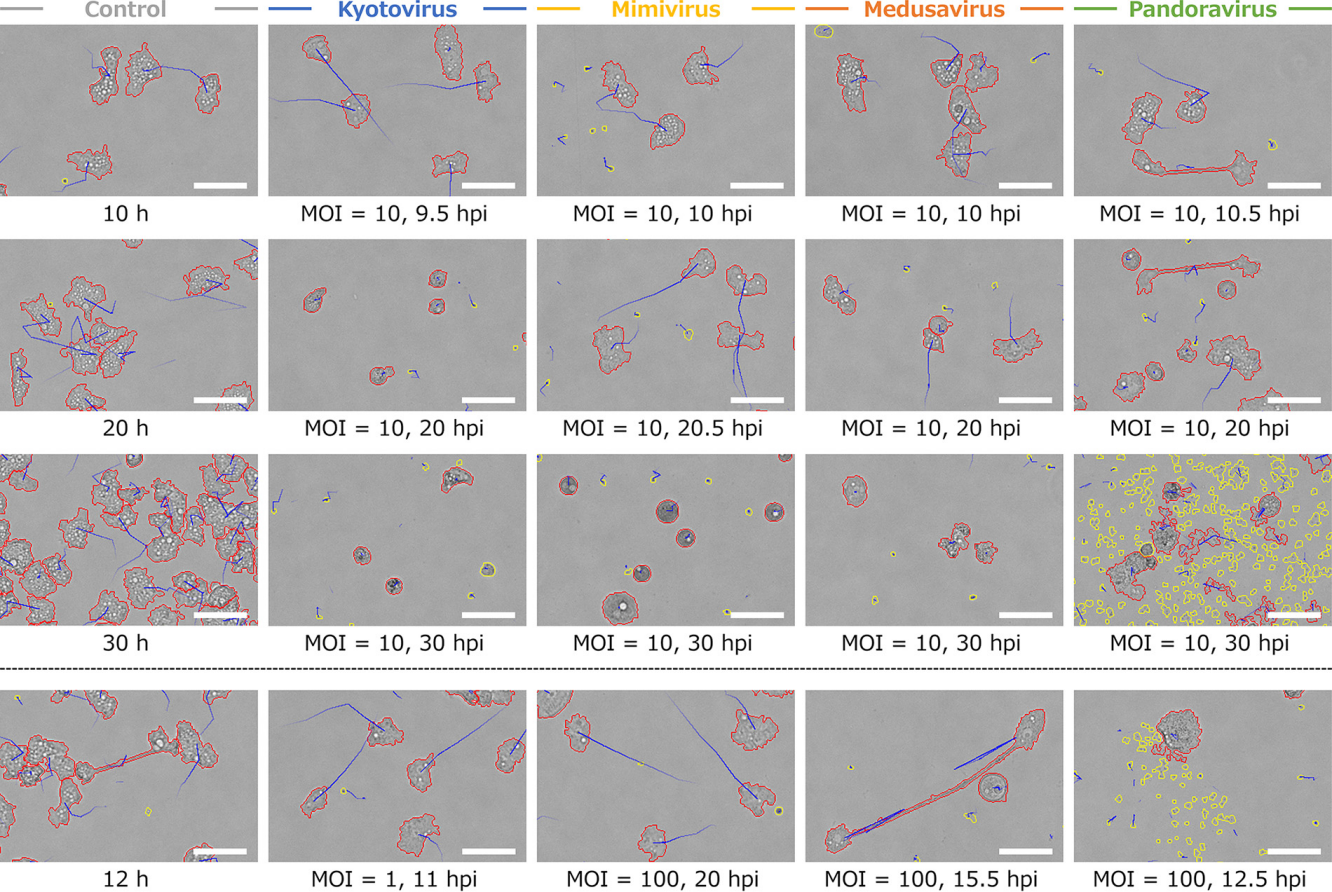
Particles detected using PKA3. The virus that infected A. castellanii is shown at the top, and the hours postinfection (hpi) and the multiplicity of infection (MOI) are shown at the bottom. The top three rows are images with MOI = 10 and the bottom row presents images with different MOIs, including characteristic particles. The control is noninfected healthy A. castellanii incubated for the time shown at the bottom of the image. The red lines indicate the outlines of particles with a size of ≥320 pixels. The yellow lines indicate the outlines of particles with a size of <320 pixels. The blue lines indicate the trajectory of the movement of the particles in the last five frames. The scale bar indicates 50 μm.

**FIG 2:**
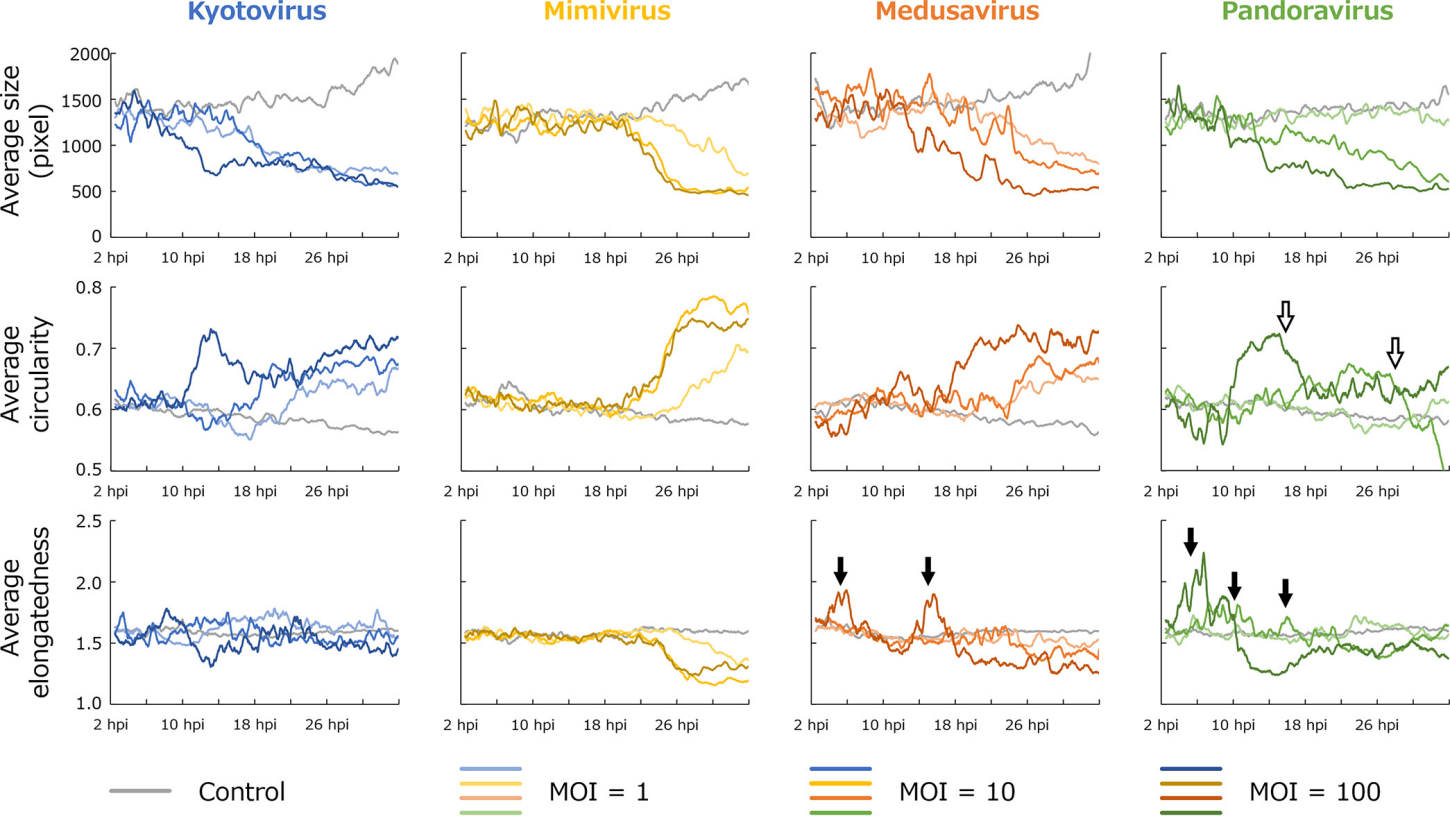
Comparison of morphological parameters. Time series changes in particle size (top), particle circularity (middle), and particle elongatedness (bottom), which was an average of all particles with a size of ≥320 pixels contained in the frame. The analysis used PKA3 of A. castellanii infected with kyotovirus (1st column), *Mimivirus shirakomae* (2nd column), medusavirus (3rd column), and *Pandoravirus japonicus* (4th column) at each multiplicity of infection (MOI) along with the results of the noninfected control. Each series is a simple moving average for 30 min (45 frames). The white arrows indicate where the average circularity decreases after increasing, and the black arrows indicate where the average extendedness is much higher than that of the control.

**FIG 3:**
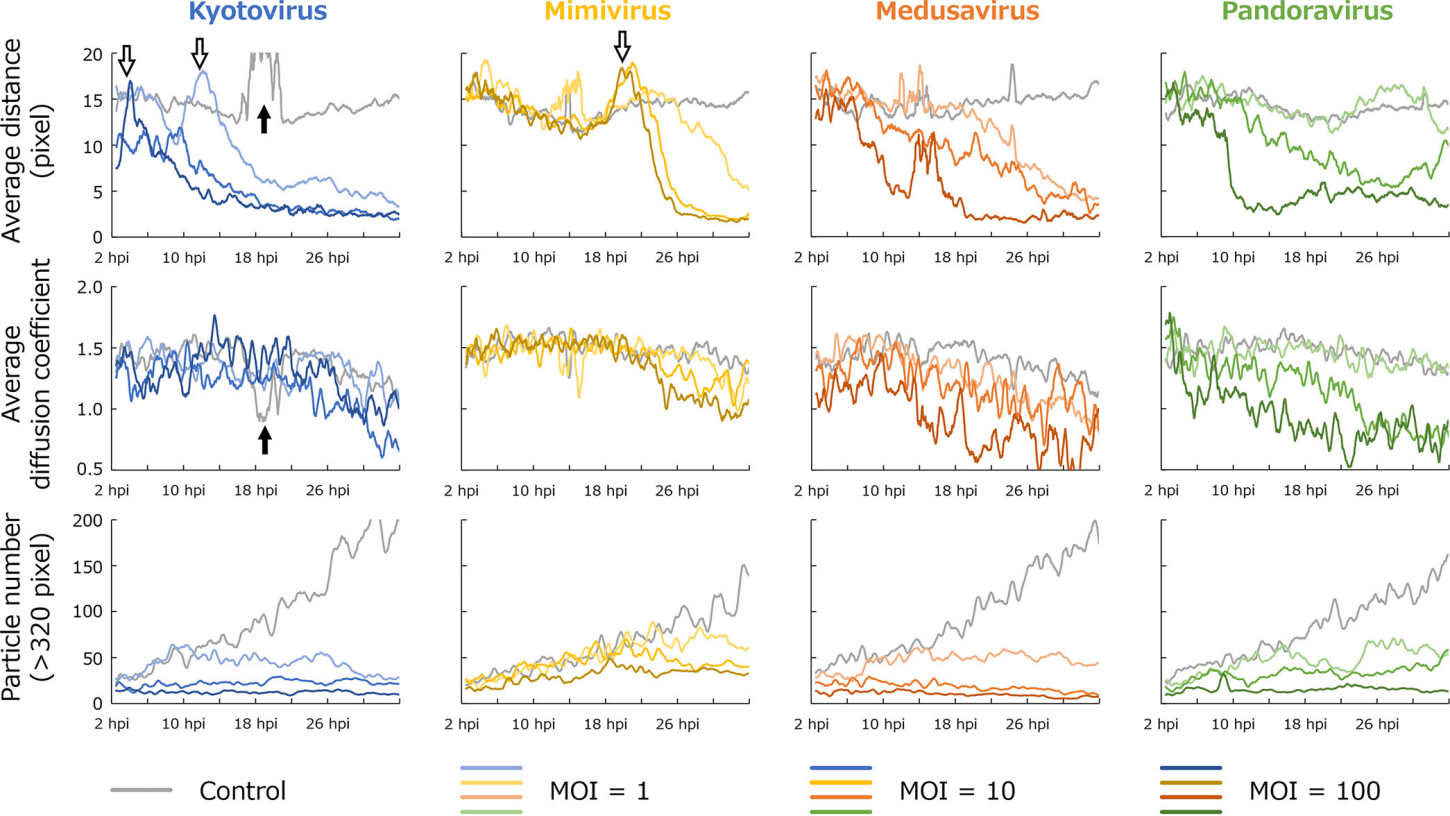
Comparison of behavioral parameters. Time series changes in average moving distance (top), average diffusion coefficient (middle), and number of particles (bottom) for all particles with a size of ≥320 pixels in the frame are shown. PKA3 analysis results of A. castellanii infected with kyotovirus (1st column), *Mimivirus shirakomae* (2nd column), medusavirus (3rd column), and *Pandoravirus japonicus* (4th column) at each multiplicity of infection (MOI) are shown along with the results of the noninfected control. Each series is a simple moving average for 30 min (45 frames). The white arrows indicate where the moving distance is higher than that of the control, and the black arrows indicate one of the changes due to the vibration of the images.

To reveal the changes that occur in A. castellanii cells due to infection by different giant viruses, time-lapse movie recording and image analysis were performed for A. castellanii cells infected with the four viruses, kyotovirus, *M. shirakomae*, medusavirus, and *P. japonicus*. Here and in the figures, mimivirus refers to *M. shirakomae*, pandoravirus refers to *P. japonicus*, and control refers to the uninfected A. castellanii cells. We made 16 time-lapse movies of A. castellanii infected with the four viruses using four different multiplicities of infection (MOIs) using a phase-contrast microscope (Movies S1 to S4). These movies were analyzed using the updated PKA3. Figure 1 shows the analyzed images that confirm the characteristic morphology or behavior of the infected and uninfected host cells at different MOIs and times. The time series changes in the morphological and behavioral parameters are shown in Fig. 2 and 3, respectively.

Uninfected amoebae (control in Fig. 1; gray series in Fig. 2 and 3) gradually increased in particle number due to cell division. The other control parameters remained almost unchanged over time until the 26-h time point. After 26 h, some of the control parameters changed due to saturation of the space at the bottom of the well due to cell growth. The average size of the particles increased because the cells overlapped partially and became a single particle that could not be distinguished (average size in Fig. 2). The diffusion coefficient decreased because the freely movable region decreased (diffusion coefficient in Fig. 3). In the control sample of the kyotovirus infection, the moving distance increased at around 18 h (black arrow in Fig. 3, top), whereas the diffusion coefficient decreased (black arrow in Fig. 3, middle), which could be due to the vibration of images. The vibration might be induced by the slippage that occurs when the field of view is moved to capture multiple wells of a microplate simultaneously by the batch capture function of the microscope. Vibration was observed in the kyotovirus control from 16 to 22 h (Movie S1, top left, 42 to 60 s), the mimivirus control at 14 h (Movie S2, top left, 36 s), mimivirus (MOI = 1) from 11 to 16 h (Movie S2, top right, 27 to 42 s), the medusavirus control from 12 to 16 h (Movie S3, top left, 30 to 42 s), medusavirus (MOI = 1) from 11 to 16 h (Movie S3, top right, 27 to 42 s), and medusavirus (MOI = 100) from 12 to 16 h (Movie S3, bottom right, 30 to 42 s). Since these changes were not due to the infection, they were not used in this study to compare the effects of the infection.

### Morphology and behavior unique to viral infection.

All virus-infected host cells were smaller in size, larger in circularity, and shorter in moving distance, and their particle number did not increase (Fig. 2 and 3). This reflects that infection-induced cell rounding, decreased motility, and inhibition of cell division are universal characteristics of cells infected with these viruses. Cell rounding and decreased motility were also confirmed in the analyzed phase-contrast microscope images as circular red lines and short blue trajectories (Fig. 1). In the kyotovirus-infected samples, the moving distance was higher than that of the control before it decreased (white arrow in Fig. 3). This is consistent with the findings of a previous study (16). In addition, a similar increase in moving distance was observed in the mimivirus-infected samples (white arrow in Fig. 3). The increase in the moving distance was also traced as long blue trajectories from the analyzed phase-contrast microscope image (9.5 h postinfection [hpi] and bottom images of kyotovirus, 10 hpi and bottom images of mimivirus in Fig. 1).

**FIG 4:**
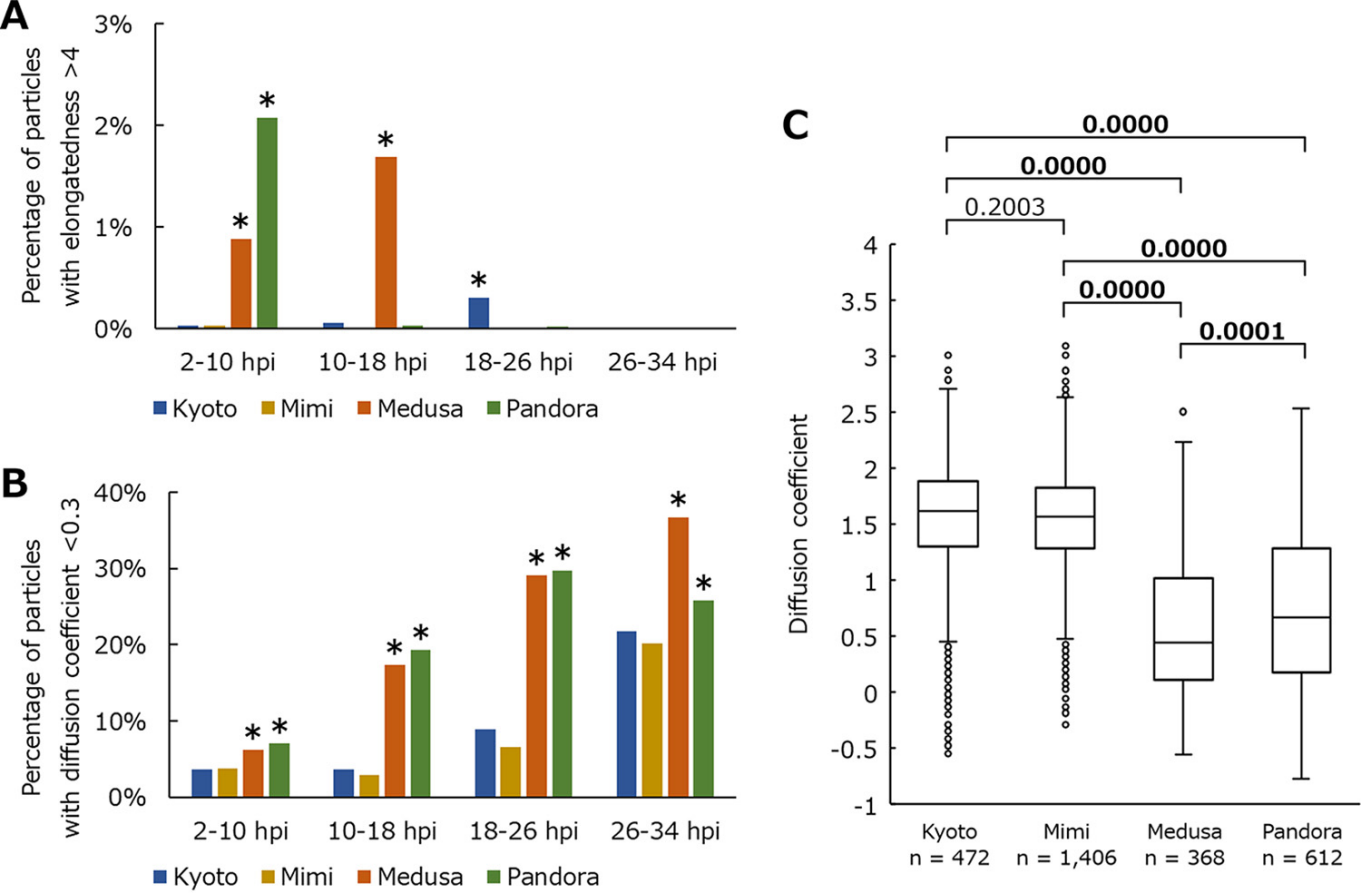
Frequency of detection of unusual particles in samples infected with viruses with a multiplicity of infection (MOI) of 100. Kyoto, Mimi, Medusa, and Pandora indicate the samples infected with kyotovirus, *Mimivirus shirakomae*, medusavirus, and *Pandoravirus japonicus*, respectively. The vertical axis in panel A represents the percentage of particles with an elongatedness of >4. The vertical axis in panel B represents the percentage of particles with a diffusion coefficient of <0.3. The asterisks in panels A and B indicate significant differences (*P* < 0.05). The boxplot in panel C represents the diffusion coefficients of all particles detected between 20 hpi and 20.5 hpi. *n* indicates the number of particles contained in each result. Values above the boxplots correspond to *P* values; bold numbers indicate significant differences (*P* < 0.05).

Medusavirus- and pandoravirus-infected samples had temporarily higher average elongatedness than their controls (black arrows in Fig. 2). Statistical analysis revealed that a significantly large number of highly elongated particles with a length of more than four times the width were observed in the early stage of medusavirus- and pandoravirus-infected cells (MOI = 100) (Fig. 4A). In the analyzed phase-contrast microscopic images, most of these highly elongated particles were amoeba cells with intercellular bridges (bottom image of medusavirus, 10.5 hpi and 20 hpi images of pandoravirus in Fig. 1). Therefore, highly elongated particles could be related to the formation of intercellular bridges, and the average elongatedness could be related to the frequency of appearance of these intercellular bridges. Intercellular bridges were also observed in uninfected and kyotovirus-infected cells (bottom image of control in Fig. 1; Movie S1) but were more frequently observed in medusavirus- and pandoravirus-infected cells (Fig. 2 and 4A). In many cases, cell division did not occur normally after the formation of an intercellular bridge.

**FIG 5:**
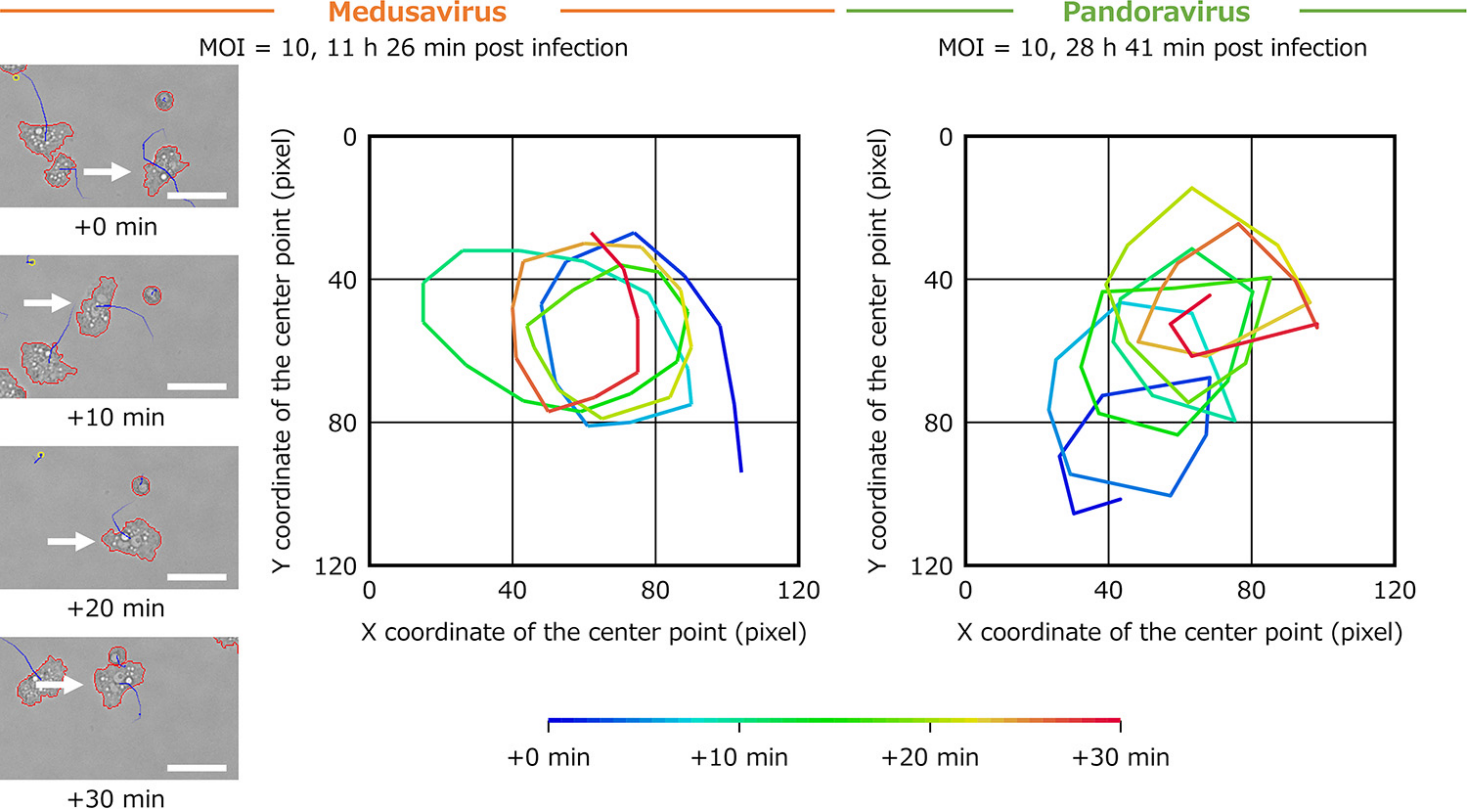
Rotational behavior of medusavirus- and pandoravirus-infected A. castellanii. The left-hand images show the particles detected using PKA3 from a fixed field of view image of a sample infected with medusavirus with a multiplicity of infection (MOI) of 10. The red lines indicate the outlines of particles with a size of ≥320 pixels. The yellow lines indicate the outlines of particles with a size of <320 pixels. The blue lines indicate the trajectories of the movement of the particles in the last five frames. The white arrow shows one particle tracked for 30 min. The scale bar indicates 50 μm. The colored lines in the right-hand panels are the 30-min trajectories of particles.

Samples infected with medusavirus and pandoravirus also had a lower diffusion coefficient than the other samples (diffusion coefficient of medusavirus and pandoravirus in Fig. 3). Statistical analysis also showed that in cells infected with these viruses (MOI = 100), the diffusion coefficient was significantly lower (Fig. 4C), and the number of particles with a low diffusion coefficient (less than 0.3) was significantly higher than that of other viruses (Fig. 4B). The long-term tracking results for such medusavirus- and pandoravirus-infected cell particles are shown in Fig. 5. These particles showed a continuous rotational behavior for more than 30 min. In Fig. 5, medusavirus-infected cells rotated about four times in 30 min, and the pandoravirus-infected cells rotated about six times in 30 min. The rotation speed varied depending on the particle, but no correlation with virus type was confirmed. In the analyzed phase-contrast microscope image at 0 min (Fig. 5), the trajectory extended in two directions because the image was taken immediately after cell division, which was prevented by the intercellular bridge formation. However, significant relationships between the intercellular bridges and the rotational behavior in medusavirus- or pandoravirus-infected cells have not been confirmed.

**FIG 6:**
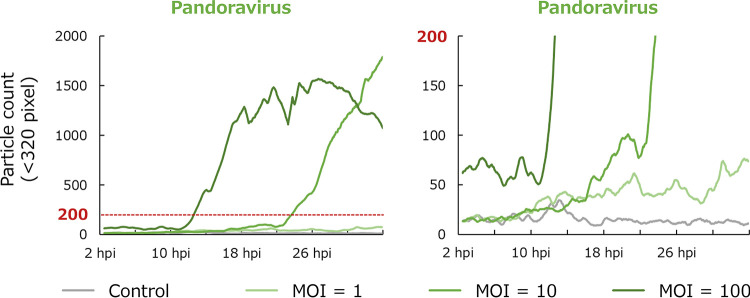
Number of small particles detected in a sample infected with *Pandoravirus japonicus*. Time series changes in the number of particles of <320 pixels in size are shown. The right-hand panel is an enlargement of the ≤200 particle count in the left-hand panel. Each series is a simple moving average for 30 min (45 frames).

Pandoravirus particles are larger than other viruses and are visible under a phase-contrast microscope; thus, they were detected using PKA3 as particles smaller than 320 pixels in size. As shown in the bottom image of pandoravirus in Fig. 1, pandoravirus particles were released from amoeba cells that gradually decreased in size. Due to this characteristic of pandoravirus particles, the infection status can be detected according to the change in the number of small particles (Fig. 6). The field of view was saturated with pandoravirus particles because, initially, the number of small particles increased gradually, and then, later, it increased rapidly. When this happened, it led to an unexpected reduction in the average circularity (white arrows in Fig. 2), which could be due to not only the morphology of the lysed amoeba cells, but also the adhesion of the pandoravirus particles to the amoebae or the particles temporarily approaching each other (30-hpi image of pandoravirus in Fig. 1). In addition, in the pandoravirus sample with an MOI of 1, inhibition of cell division was detected even though there was almost no change in the other parameters (particle number of pandoravirus in Fig. 3).

### Morphologically or behaviorally nonstandard particles.

**FIG 7:**
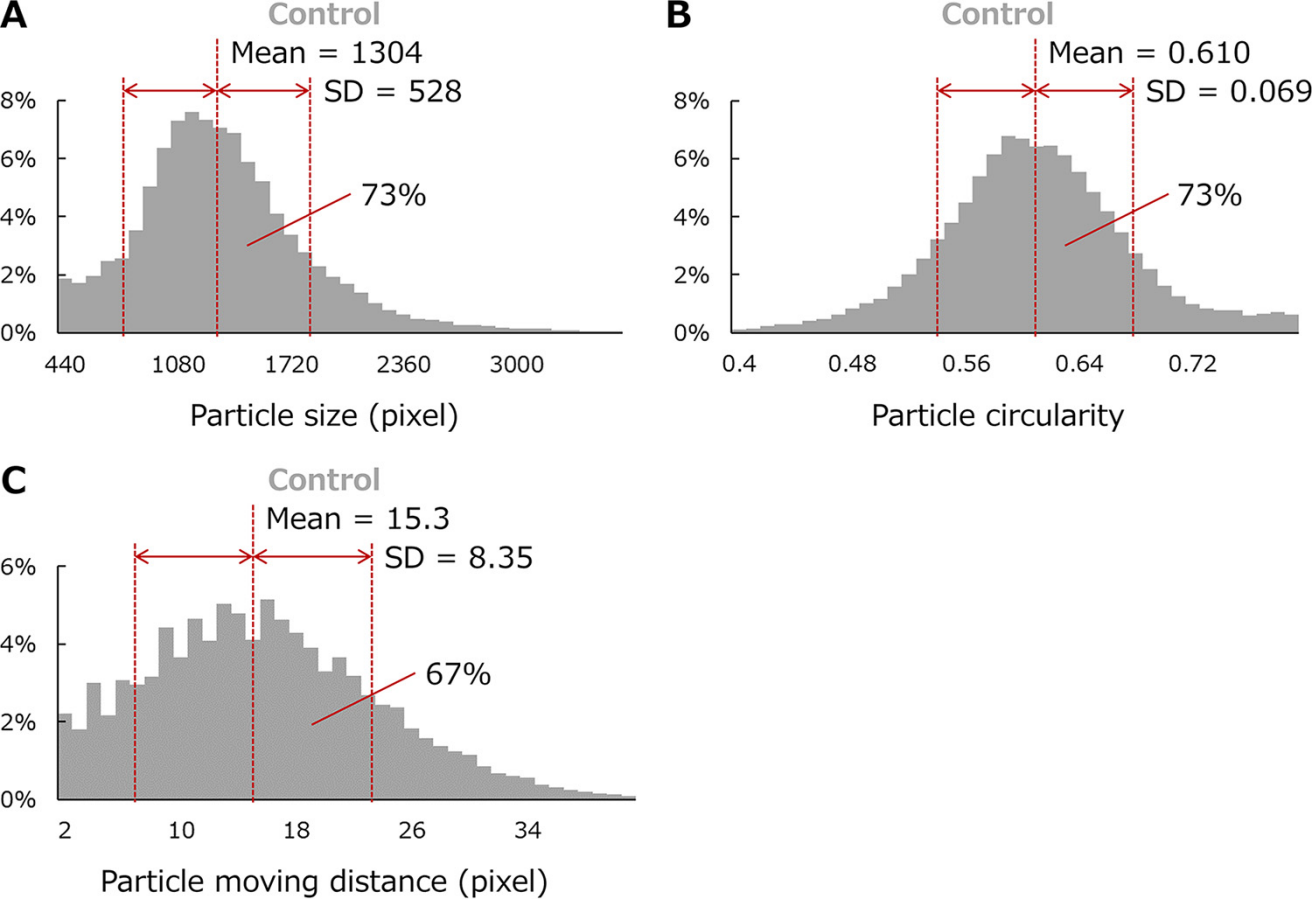
Definition of the range of each parameter of typical A. castellanii cells. The distribution of size (A), roundness (B), and moving distance (C) of 43,368 particles, which are part of the particles detected using PKA3 from the control sample, are shown. Mean indicates the simple arithmetic mean, and SD indicates the standard deviation. The percentage of particles in the mean ± 1 SD range is shown in each figure.

**FIG 8:**
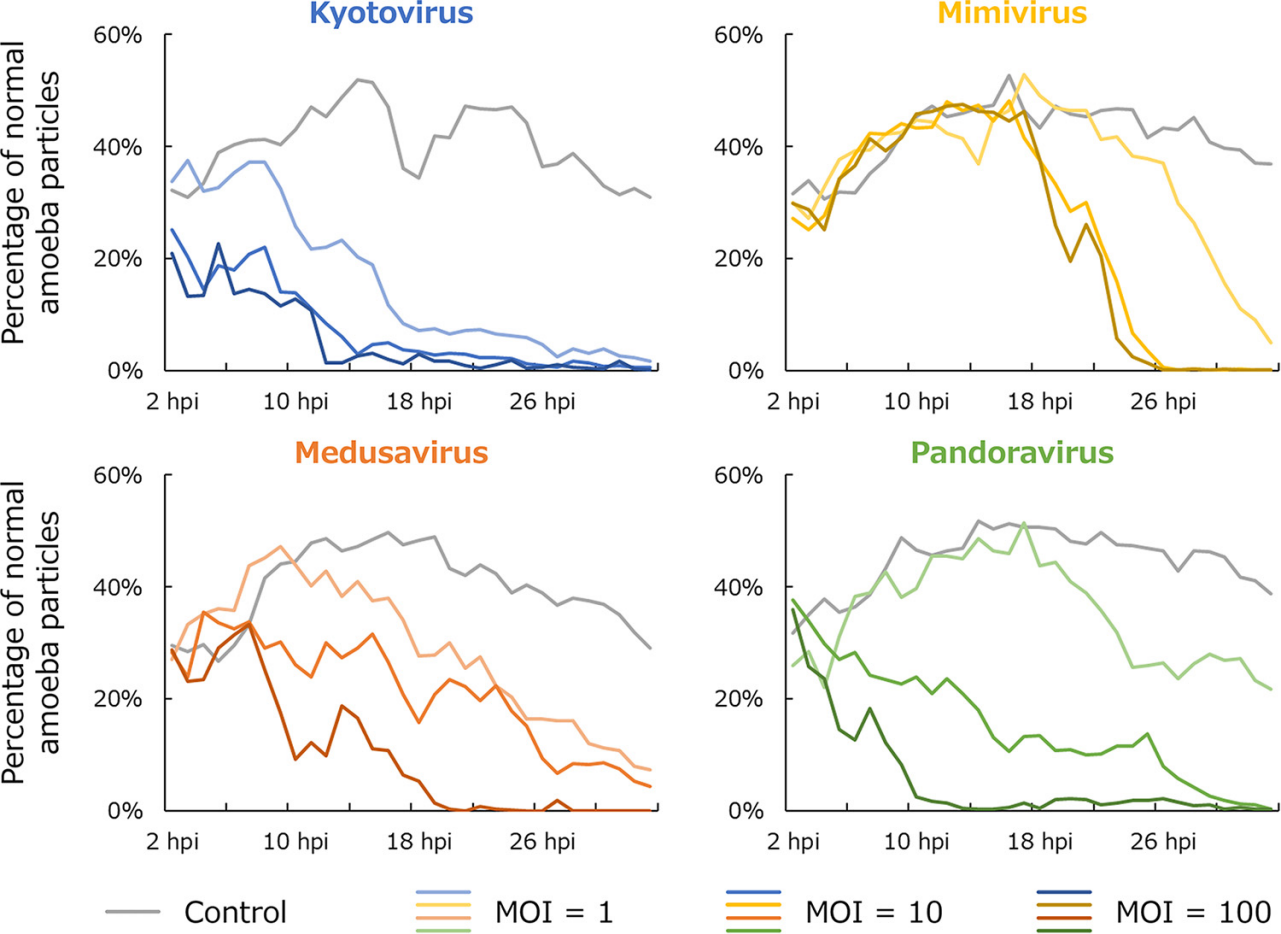
Time series changes in the proportion of particles with typical normal A. castellanii cell parameters. A particle with typical normal A. castellanii cell parameters is defined as a particle in which all three parameters (i.e., size, roundness, and moving distance) fall within the mean ± 1 SD range. The results of A. castellanii infected with kyotovirus (top left), *Mimivirus shirakomae* (top right), medusavirus (bottom left), and *Pandoravirus japonicus* (bottom right) at each multiplicity of infection (MOI) are shown along with the results of the noninfected control. Each series is a 1-h average.

The effects of infection were examined according to the number of particles with morphological or behavioral parameters that deviated from those of standard amoebae. The range of each parameter in typical amoeba cells was defined using 43,368 particles detected between 4 and 14 h from a control sample of pandoravirus-infected microplates (Fig. 7). A typical amoeba cell was defined as having three parameters within the following range: particle size (777 to 1,832 pixels), circularity (0.541 to 0.678), and moving distance (6.95 to 23.72 pixels). The percentages of typical amoeba particles detected in each sample are shown in Fig. 8.

**FIG 9:**
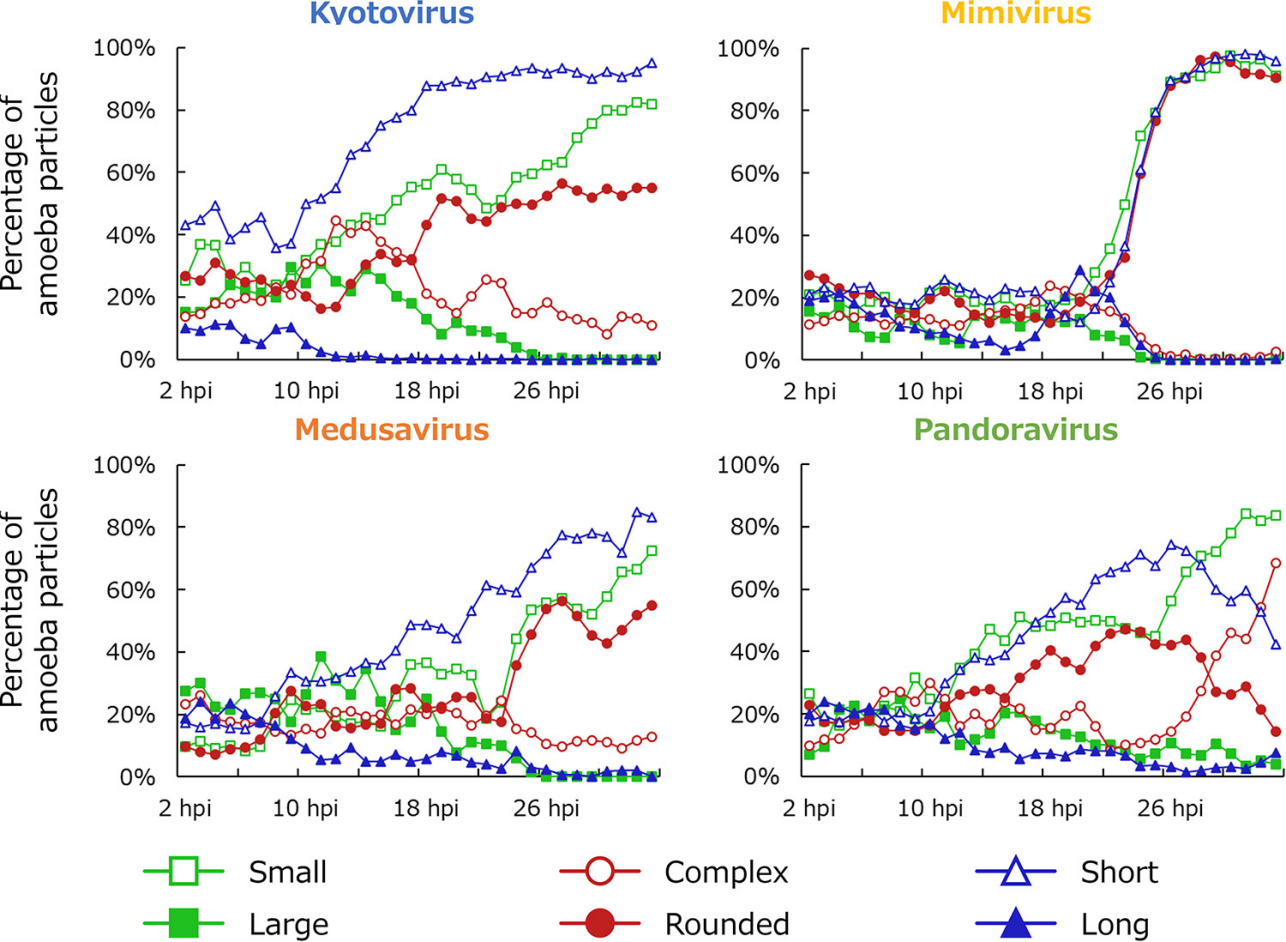
Time series changes in the proportion of anomalous particles by a particular parameter. The results of A. castellanii infected with kyotovirus (top left), *Mimivirus shirakomae* (top right), medusavirus (bottom left), and *Pandoravirus japonicus* (bottom right) with a multiplicity of infection (MOI) of 10 are shown. Small, complex, and short indicate the proportion of particles whose size, circularity, and moving distance, respectively, are smaller than the mean – 1 SD. Large, rounded, and long indicate the proportion of particles whose size, circularity, and moving distance, respectively, are greater than mean + 1 SD. Each series is a 1-h average.

After infection, the proportion of typical cells decreased with time and decreased faster with higher MOI. In the kyotovirus-infected amoebae with high MOI, the proportion of typical amoebae began to decrease before 2 h postinfection (hpi) and was almost nonexistent at 12 hpi. In the kyotovirus-infected amoebae with low MOI, there were almost no typical amoebae left at 18 hpi, although there was almost no decrease in those infected with other viruses. In the mimivirus-infected amoebae, the proportion of typical amoebae did not decrease until around 18 hpi, even with a high MOI. After that, it decreased sharply, and 8 h after the start of the decrease, the proportion of typical amoebae became almost 0. According to the data shown in Fig. 9, it was clear that deviation of all parameters occurred at the same time in the mimivirus-infected amoebae. In the samples infected with other viruses, cells with shorter moving distances appeared earlier than cells that were anomalous due to other causes.

## DISCUSSION

In this study, we used time-lapse video imaging and PKA3 for analyzing amoeba cells infected with four types of giant viruses to reveal the morphological and behavioral differences of host cells during the process of infection. The results showed a temporary increase in the moving distance (kyotovirus and mimivirus), particles with intercellular bridges or rotational behavior (medusavirus and pandoravirus), an early decrease in the number of typical amoebae with low MOI (kyotovirus), and coordinated changes in morphology and behavior with high MOI (mimivirus).

The results of this study on moving distance in kyotovirus-infected cells were consistent with the results of a previous study with a temporary increase in the moving distance of kyotovirus-infected A. castellanii cells (16). In addition, this study revealed that a similar increase in moving distance also occurred with mimivirus infection (white arrows in Fig. 3).

The intercellular bridges were frequently observed in the medusavirus- and pandoravirus-infected cells, but not in the mimivirus-infected cells (Fig. 1; Movie S1). These intercellular bridges were similar to those described in a previous study of uninfected *Acanthamoeba* cells (20). Intercellular bridges are often observed during the division of multinucleated cells and amitosis (20). Moreover, multinuclearity is enhanced in A. castellanii during unfavorable growth conditions (21). Therefore, we considered that intercellular bridge formation may be a common defense mechanism of A. castellanii against the unfavorable conditions in the nucleus or division. Nonviral factors also cause such unfavorable conditions, but medusavirus or pandoravirus infections could be more potent causes. In Fig. 1, a black structure is visible in the nuclear envelope of only one cell of the intercellular bridge. Although it is possible that there is a difference in the states of the two nuclei, significant results could not be obtained because both nuclei were in focus during time-lapse photography only at a few instances. These can be further elucidated by manual focusing and imaging or by staining the nuclei with nuclear stains such as 4′,6-diamidino-2-phenylindole (DAPI).

Time-lapse movies have also shown similar changes between the medusavirus- and pandoravirus-infected cells (Movies S3 and S4). We observed that these viruses have the common characteristic of not only causing intercellular bridges, but also of causing rotating behavior (Fig. 5) in host cells. Both viruses replicate using the host nuclear machinery (5, 8), suggesting that this could affect the host cell functions and result in abnormal behaviors during the infection, which is common in both viruses. *Marseilleviridae* family viruses also use the host nuclear machinery, but in this case, the nuclear machinery is transiently recruited to their site of viral replication, and then the nucleus recovers its integrity (22). This contrasts with the fact that DNA replication of medusavirus takes place in the host’s nucleus (8), and pandoravirus causes confusion in the host nucleus (5). A biochemical approach to the effects of viruses on the nuclear function of host cells may provide new insights into the interactions between viruses and host cells.

With low MOI, the morphological changes in A. castellanii cells infected with kyotovirus progressed faster than those of the other viruses; this is consistent with the results of a previous study that used flow cytometry for analysis (13). *Marseilleviridae* family viruses have been suggested to form vesicles or groups containing multiple viruses and being of a size that amoebae can phagocytose (23). Thus, it is unlikely that all the cells were infected at the same time with kyotovirus with low MOI. Therefore, the fact that most cells infected with kyotovirus (MOI = 1) did not have the typical parameters at 18 hpi shows a faster change than expected. These results suggest that the replication cycle of kyotovirus is unusually more rapid than that of mimivirus, pandoravirus, or medusavirus. This may be related to the unique ability of *Marseilleviridae* family viruses to form vesicles containing multiple viruses upon infection and release (23). Previous study has also demonstrated that the faustovirus-infected Vermamoeba vermiformis released the encystment factor, which led to the encystment of the uninfected amoebae (24). Analysis of compounds in the culture medium of kyotovirus-infected cells may reveal why this virus causes CPE faster than expected.

The starting time of cell morphology and behavior changes observed in the mimivirus-infected cells with low MOI was not considerably different from that of medusavirus- and pandoravirus-infected cells. As the MOI increased, the starting time of changes in medusavirus- and pandoravirus-infected cells occurred earlier. However, the starting time of changes in mimivirus-infected cells was fixed regardless of the MOI. Instead, the higher the MOI, the shorter the period of time for completion of the change. For example, the average size of medusavirus-infected cells with an MOI of 100 gradually decreased from approximately 12 hpi to 26 hpi (Fig. 2). On the other hand, the average size of the mimivirus-infected cells rapidly decreased from approximately 20 hpi to 26 hpi (Fig. 2). Furthermore, in the mimivirus-infected cells with an MOI of 10, the changes in three parameters (size, circularity, and moving distance) started and finished simultaneously (Fig. 9). This is consistent with the results of a previous study that the change in the circularity of Acanthamoeba polyphaga infected with Acanthamoeba polyphaga *mimivirus* (APMV) was completed in just 4 h after the change began (12). These results indicate that the mimivirus initially allowed the host cell to behave normally, even with a high MOI, and then caused a rapid CPE. This suggests that mimivirus has a unique infection strategy that increases the range and efficiency of infection due to allowing host cells to actively migrate and release virus particles far away from the place of infection. Further research, such as observation in a wider area for host cells in this state or measurement of viral titers in the culture medium, is required in the future.

Thus, it has been shown that there is a diversity in host cell CPEs during the giant virus infection process. It may be possible to identify the giant virus using the morphological and behavioral parameters of the infection process, as it was possible to identify the virus using a flow cytometer in a previous study (13). In particular, the finding that *M. shirakomae* has a unique and fixed timing of CPE, which may be a general feature of *Mimiviridae* family viruses, suggests that a wide variety of giant virus infection strategies are difficult to unravel without observing the infection process of living cells. However, the results of this study are limited to the morphological and behavioral changes of the host cells based on microscopic observations. The host cell response to viral infection may be due to a spreading strategy of the viruses or an antiviral strategy of amoebae, which could not be confirmed by the results of this study. *Mimiviridae* family viruses have been reported to have genes related to actin (25), which *Acanthamoeba* utilizes to maintain its cell shape and form pseudopodia for migration. The metagenomic analysis of giant viruses has also revealed a variety of metabolic genes that have been suggested to change the host cell’s behavior by altering its nutritional demands (26). Molecular biological studies, including alterations of such genes, and time series omics analysis, are needed to further elucidate the function of the virus and virus-host interactions.

In conclusion, we quantitatively revealed the diversity of CPEs in the host cell, A. castellanii, during the infection process of four giant viruses. These findings could be important for understanding the interaction between giant viruses and host cells. Furthermore, our results shown here suggest that kinetic analysis of virus-infected amoeba cells using PKA3 reveals the infection strategies of each giant virus, which have not been elucidated yet. In the future, it will be necessary to elucidate the biochemical changes that occur during these processes. Also, the analysis of morphological parameters by PKA3 is limited to cellular outlines. Analyzing the unclear and complex cytoplasm may reveal unknown changes in host cells due to viral infections. In the future, it will be necessary to take a new approach considering both a microscopy method for clear cytoplasmic imaging and algorithms for analyzing complex structures. In addition, the image analysis technique using PKA3 could be applied to many other giant viruses that infect A. castellanii. Morphological and behavioral analyses in host cells infected with these viruses may reveal further unknown CPE processes.

## MATERIALS AND METHODS

### Culturing A. castellanii cells.

Acanthamoeba castellanii (Douglas) Neff (ATCC 30010^TM^) cells were purchased from the American Type Culture Collection (ATCC; Manassas, VA, USA) and cultured in proteose-peptone-yeast extract-glucose (PYG) medium at 26°C according to the ATCC protocol as described in previous studies (8, 10, 27).

### Mimivirus, kyotovirus, medusavirus, and pandoravirus.

*Mimivirus shirakomae* of the *Mimiviridae* family, kyotovirus of the *Marseilleviridae* family, medusavirus, and *Pandoravirus japonicus* of the *Pandoraviridae* family were isolated in our laboratory from water samples collected in Japan from the Tatakai River in Kyoto (kyotovirus), the Shirakoma Ponds in Nagano (*M. shirakomae*), Japanese hot springs (medusavirus), and the Sabaishi River in Niigata (*P. japonicus*). Isolation of the viruses was performed by screening A. castellanii cells showing CPE in 96-well plates (8, 10, 18, 19). Each virus propagated using A. castellanii cells in one or several 75-cm^2^ flasks was stored in phosphate-buffered saline at 4°C as previously described (8, 10, 18, 19). The MOI of each virus was calculated as previously described (10). Briefly, 90 μl of PYG medium with A. castellanii cells was added to each well of 96-well microplates. Thereafter, 10 μl of PYG medium containing 10-fold serially diluted virus in 10 steps was added to each well. The culture medium was incubated at 26°C for 4 days and then observed under a microscope. The virus titer was calculated using a 50% tissue culture infective dose (TCID_50_) calculator v.2.1 (Marco Binder, Department of Infectious Diseases, Molecular Virology, Heidelberg University).

### Obtaining time-lapse images.

Time-lapse phase-contrast movies of A. castellanii cells were obtained using an all-in-one fluorescence microscope (BZ-X800/X810; Keyence Co., Osaka, Japan) with a ×20 lens objective (CFI Plan Fluor DL ×20; Nikon Instech, Tokyo, Japan). This microscope allows time-lapse microscopic imaging in the bright-field, phase-contrast, and fluorescence modes. Additionally, images of multiple wells of a microplate were obtained simultaneously. A Precision 3430 desktop computer (Dell, Inc., Round Rock, TX, USA) with a CPU (Xeon E-2124; Intel Co., Santa Clara, CA, USA), 16.0 GB memory, and GPU (Quadro P400; Nvidia Co., Santa Clara, CA, USA) was used to operate the microscope.

As samples for these movies, 50,000 amoebae in PYG medium were prepared. The number of amoeba cells was determined using a disposable cell counter (WC2-100; WakenBtech Co., Kyoto, Japan). Virus-containing PYG medium of a volume suitable for the desired MOI (1, 10, or 100) was added to these samples. Finally, these samples were diluted to a total volume of 2.5 ml, resulting in an A. castellanii concentration of 20,000 cells/ml. Then, 2 ml of each sample was taken in each well of the 12-well microplates. Three different MOI (1, 10, and 100) samples of the same virus and one with the same A. castellanii concentration (control) in PYG medium were added to the wells in a 12-well microplate and kept under the microscope for 2 h for stability. Subsequently, four samples in one microplate were imaged at the same time using the parallel imaging function of the microscope. Each frame in the time-lapse movies was an image of 960 by 720 pixels with 256 grayscale tones. A total of 2,880 phase-contrast images were obtained from each well of the microplate by capturing images every 40 s for 32 h.

### Updating PKA3.

To detect larger particles from phase-contrast microscope images, we updated PKA3 (16), which is written in C/C++ and is accelerated by a GPU with OpenCL (28). A particle was considered a mass of area that cannot be regarded as background; thus, it may be either a single living amoeba cell, a single dead amoeba cell, an aggregate of amoeba cells, or debris. The previous algorithm was fast but could not detect large particles because the required memory depended on the size of the detected particles. This was not an issue when observing with the ×4 lens objective, but it was an issue when observing with the ×20 lens objective. The new algorithm detects particles using the difference in intensity, similarly to the previous one, and the required memory is constant regardless of particle size, but the calculation takes more than twice as long as before.

Like the previous algorithm, the output of the updated algorithm is in the form of the number of particles in the frame and the size, circularity, moving distance, and moving direction of each particle. The circularity was calculated using the following equation: 4 π *S*/*L*^2^, where S is the area of the particle, π is the circumference ratio, and L is the perimeter of the particle (29). In addition, the new algorithm provided output in the form of the particle’s unique ID, elongatedness, diffusion coefficient, Voronoi cell size, and Delaunay edge length. A unique ID was assigned to a series of particles if it could be tracked without cell division or aggregation. Elongatedness is the value obtained by dividing the length of the minimum bounding rectangle at the widest angle by the width of the minimum bounding rectangle at the narrowest angle (30). The diffusion coefficient is a power index obtained by the power approximation of the plot of the mean squared displacement for the last 30 min (31). If the diffusion coefficient is greater than 1, the particle movement is more diffusive than normal diffusion. Voronoi and Delaunay diagrams were drawn using the center points of each particle to calculate the Voronoi cell size and Delaunay edge length, respectively. The Delaunay diagram can be used as an index to quantify the alignment of particles in image analysis (32).

A Microsoft Surface Laptop 3 computer (Microsoft Co., Redmond, WA, USA) with a CPU (Ryzen 5 3580U; Advanced Micro Devices, Inc., Santa Clara, CA, USA), 8.0 GB memory, and GPU (Radeon Vega 9; Advanced Micro Devices Inc.) was used to develop the program and analyze the images. With this setup, an average of approximately 0.5 s was required to analyze each image of 960 by 720 pixels generated in this study.

### Statistical analysis.

The significance of each result was analyzed using the chi-square test (Fig. 4A and B) and the Mann-Whitney U two-sided test (Fig. 4C). The *n* and *P* values of the Mann-Whitney U test are shown in each figure. Microsoft Excel (Microsoft Co., Redmond, WA, USA) was used to create the graphs and calculate the standard deviations.

### Data availability.

The microscopic images taken in this study, source code of PKA3, and the file output by analysis using PKA3 are available at https://pkaaa2021.z11.web.core.windows.net/.
