# Supplementary material for: Kinetic Analysis of Acanthamoeba castellanii Infected with Giant Viruses Quantitatively Revealed Process of Morphological and Behavioral Changes in Host Cells
Source: Microbiol Spectr. 2021 Aug 25;9(1):10.1128/spectrum.00368-21. doi: 10.1128/spectrum.00368-21 (PMC8552732; doi:10.1128/spectrum.00368-21)
Supplement: SUPPLEMENTAL FILE 1 — Supplemental material. Download SPECTRUM00368-21_Supp_1_seq2.pdf, PDF file, 0.1 MB [file spectrum00368-21_supp_1_seq2.pdf]

Supplementary Materials

Supplementary Movie 1 Time-lapse phase-contrast microscopic movie of *A. castellanii* infected with kyotovirus with MOI = 1 (top right), MOI = 10 (bottom left) and MOI = 100 (bottom right) and uninfected *A. castellanii* as a control (top left). The 3 s of this movie were 1 h in real time.

Supplementary Movie 2 Time-lapse phase-contrast microscopic movie of *A. castellanii* infected with *Mimivirus shirakomae* with MOI = 1 (top right), MOI = 10 (bottom left) and MOI = 100 (bottom right) and uninfected *A. castellanii* as a control (top left). The 3 s of this movie were 1 h in real time.

Supplementary Movie 3 Time-lapse phase-contrast microscopic movie of *A. castellanii* infected with medusavirus with MOI = 1 (top right), MOI = 10 (bottom left) and MOI = 100 (bottom right) and uninfected *A. castellanii* as a control (top left). The 3 s of this movie were 1 h in real time.

Supplementary Movie 4 Time-lapse phase-contrast microscopic movie of *A. castellanii* infected with *Pandoravirus japonicus* with MOI = 1 (top right), MOI = 10 (bottom left) and MOI = 100 (bottom right) and uninfected *A. castellanii* as a control (top left). The 3 s of this movie were 1 h in real time.
